# CytoKavosh: A Cytoscape Plug-In for Finding Network Motifs in Large Biological Networks

**DOI:** 10.1371/journal.pone.0043287

**Published:** 2012-08-29

**Authors:** Ali Masoudi-Nejad, Mitra Ansariola, Zahra Razaghi Moghadam Kashani, Ali Salehzadeh-Yazdi, Sahand Khakabimamaghani

**Affiliations:** Laboratory of Systems Biology and Bioinformatics (LBB), Institute of Biochemistry and Biophysics, University of Tehran, Tehran, Iran; University of Westminster, United Kingdom

## Abstract

Network motifs are small connected sub-graphs that have recently gathered much attention to discover structural behaviors of large and complex networks. Finding motifs with any size is one of the most important problems in complex and large networks. It needs fast and reliable algorithms and tools for achieving this purpose. CytoKavosh is one of the best choices for finding motifs with any given size in any complex network. It relies on a fast algorithm, Kavosh, which makes it faster than other existing tools. Kavosh algorithm applies some well known algorithmic features and includes tricky aspects, which make it an efficient algorithm in this field. CytoKavosh is a Cytoscape plug-in which supports us in finding motifs of given size in a network that is formerly loaded into the Cytoscape work-space (directed or undirected). High performance of CytoKavosh is achieved by dynamically linking highly optimized functions of Kavosh's C++ to the Cytoscape Java program, which makes this plug-in suitable for analyzing large biological networks. Some significant attributes of CytoKavosh is efficiency in time usage and memory and having no limitation related to the implementation in motif size. CytoKavosh is implemented in a visual environment Cytoscape that is convenient for the users to interact and create visual options to analyze the structural behavior of a network. This plug-in can work on any given network and is very simple to use and generates graphical results of discovered motifs with any required details. There is no specific Cytoscape plug-in, specific for finding the network motifs, based on original concept. So, we have introduced for the first time, CytoKavosh as the first plug-in, and we hope that this plug-in can be improved to cover other options to make it the best motif-analyzing tool.

## Introduction

The network concept is widely used to analyze and predict the dynamics of a complex system [1]. Biological processes are often represented in the form of networks such as: gene regulation, signal transduction, protein interaction and metabolic networks. The study of biological networks, their modeling, analysis and visualization, is an important task in the field of life science. Computational tools help biologists through this difficult study. An understanding of these networks is essential to make the biological sense of much of the complex data that is now being generated [2]. When we talk about the networks, the complex system is perceived as a set of interacting elements (nodes, vertices), which are bound together by links (contacts, edges, interactions). In usual networks (graphs), links represent the interactions between element pairs [3]. Graphs are used for representing and modeling the networks to analyze them in a scientific and formal manner.

The main attributes of the biological networks are their complexity and vast amount of data, so extracting the meaningful data from them needs powerful and accurate methods. Motifs are the building blocks of complex networks, providing a bridge between local vertex-related properties and global functional properties of networks. Motif analysis in the network is notably important because they may reflect functional properties [1].

A motif is a small connected graph expressed as G. The size of a motif is represented by the number of vertices (nodes) [4]. Different methods and stand-alone tools have been developed for analyzing the network motifs: Mfinder [5], MAVisto [6], NeMoFinder [7], Grochow-Kellis [8], Color-coding approach [9], G-Tries [10], FANMOD [11], Kavosh [12] and MODA [13]. Mfinder implements two kinds of motif-finding algorithms: a full enumeration and a sampling method. The sampling protocol is the faster one that assigns probability values to the motifs to be identified, and infers frequencies from these values. It is also a tool without the option of a visual presentation and the results are only provided in the format of a text. For achieving best analysis results from network analysis tools, undoubtedly, visual capability of the analysis tools is an important factor. MAVisto is a visual tool, but in comparison with other programs, it needs more time and in the matter of time it has limitation in motif size. NeMoFinder utilizes frequent size-n trees to partition the input network into a collection of size-n graphs, afterward finds frequent size-n sub-graphs through expansion of frequent trees edge-by-edge until getting a complete size-n graph *K_n_*. The algorithm finds network motifs from undirected network and is not limited to extracting just induced sub-graphs. Furthermore, NeMoFinder is an exact enumeration algorithm and is not based on a sampling method. Grochow and Kellis algorithm is based on a motif-centric approach, which means that the frequency of a given sub-graph, called query graph, is exhaustively determined through searching for all possible mappings from the query graph into the larger network. Ribeiro proposed a novel data structure for storing a collection of sub-graphs, called G-trie. This data structure that is conceptually akin to prefix tree, store sub-graphs due to their structures and find occurrences of each sub-graphs in a larger graph. One of the noticeable aspects of this data structure is that coming to the network motif discovery, the sub-graphs in the main network are needed to be evaluated. So, there is no need to find the ones in random network which are not in the main network. FANMOD algorithm is one of the best among others, in regard to the computational time. But it can handle sub-graphs, consisting of a maximum of eight vertices. MODA and Kavosh are two non-visual algorithms having good performances. The Kavosh plug-in is the best tool, according to the time and memory usage, among all and has no limitation in the size of motifs studied by this program [12].

Here, we introduce CytoKavosh as the first network motif finder plug-in for Cytoscape, which uses all Kavosh features and strengthen the studies of finding network motifs based on Milo [1]. CytoKavosh finds network motifs with less memory and CPU time in comparison with other existing tools (for more information see reference [12]) and has implemented in a known graphical environment that is familiar among the analysts of biological-networks. The results of CytoKavosh can be used in-line with other analysis tools in the integrated software to achieve the desired goals.

## Methods

CytoKavosh is implemented in Java that uses Cytoscape API. It uses Kavosh algorithm, which makes it faster than similar programs, based on other algorithms. CytoKavosh is suitable to detect network motif in both the directed and undirected networks. The main idea of the enumeration is based on Kavosh [12] and is similar to the FANMOD and MODA algorithms, which first find all k-sized sub-graphs that a particular vertex participated in, then remove that vertex, and subsequently repeat this process for the remaining nodes [12]. By using this idea, we gradually reduce the size of input network that leads us to better performance..For applying the idea of removing totally studied vertices, and also for simplifying the implementation, each vertex in the input network is given an integer label as its identification. We use these labels to apply necessary restrictions. Building an implicit tree according to some restrictions, that will be discussed later in details, causes the improvement in both time and memory usage. The tree structure with its restrictions ensures that each individual sub-graph is enumerated only once that leads us to an efficient solution.

For counting the sub-graphs of size *k* that include a particular vertex, implicit trees with maximum depth of *k*, rooted at this vertex and based on neighborhood relationship are implicitly built. Descendents of each vertex include both incoming and outgoing adjacent nodes. To descend the tree, a descendent is chosen at each level with the restriction that a particular descendent can be included only if it has not been included at any upper level. After having descended to the lowest possible level, the tree is again ascended and the process is repeated with the stipulation that vertices are visited in earlier descending paths that is now considered as the unvisited vertices. A final restriction in building the trees is that all descendents within a particular tree must have numerical labels larger than the label of the root of a tree. This restriction simulates the process of removing the vertices which are formerly considered.

The protocol for extracting sub-graphs makes use of the composition operation of an integer. For the extraction of sub-graphs of size *k*, all possible compositions of the integer *k-*1 must be considered. This composition is a pattern for choosing vertices at each level of the implicit tree. Clearly choosing the root as one of the sub-graph's vertices is unavoidable, so in order to extract a *k*-size sub-graph, *k*-1 vertices are remained. The compositions of *k-*1 consist of all possible manners of expressing *k-*1 as the sum of positive integers. Summations in which the order of the summands differs are considered as distinct. A composition can be expressed as *k*_2_, *k*_3_, …, *k_m_* where *k*_2_+*k*_3_+…+*k_m_* = *k-*1. To count sub-graphs based on the composition, *k_i_*nodes are selected from the *i*-th level of the tree that represents the nodes of the sub-graphs (*i* = 2, 3, …, *m*). The *k-*1 selected nodes, along with the node at the root, define a sub-graph of size *k* within the network.

**Figure 1:**
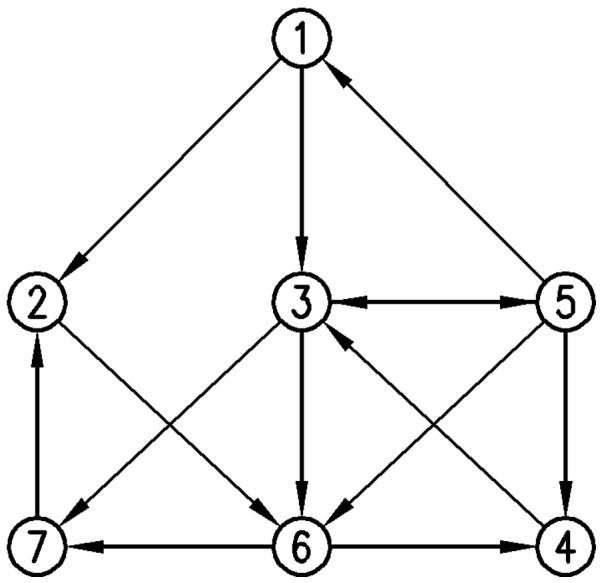
An instance of a network.

**Figure 2:**
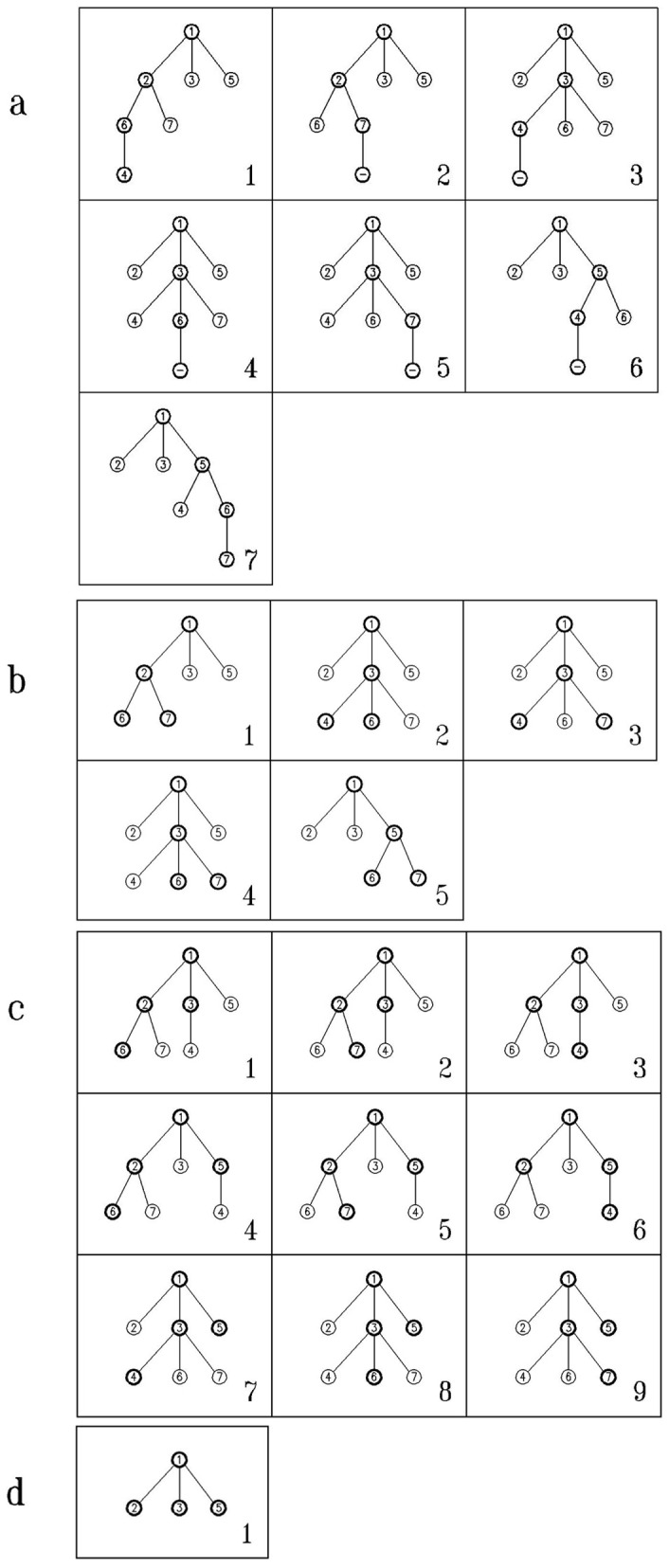
The implicit built trees rooted at vertex 1 of size 4 for network in **Figure 1**. (a) Trees built according to (1,1,1) pattern. According to this pattern, after selecting vertex 1 in root, one of its neighbors must be selected, so the second selected vertex is vertex 2. Continuing the selecting process, one of the neighbors of the vertex 2 (vertex 6) and after that vertex 4 is selected. All chosen vertices are shown by specified circles in these figures. (b) Trees built according to (1,2) pattern. (c) Trees built according to (2,1) pattern. (d) Tree built according to (3) pattern.

To clarify the algorithm, it is necessary to mention that for a particular level *i*, it is possible to have *k_i_*<*n_i_*, where *n_i_* is the number of nodes present at the level *i*. At level *i*, *C*(*n_i_*, *k_i_*) (*C*(*n*, *k*) is the number of different combinations of *k* through *n* elements) different selections of nodes needed to be considered. Here, by using the “revolving door order” algorithm [14], all combinations containing *k_i_* from *n_i_* nodes are selected. The “revolving door ordering” algorithm is considered as well-known and efficient algorithm for generating combinations [15]. This step is described by an example on a given graph shown in Figure 1. For this graph, all 4-size sub-graphs containing the vertex 1, are going to be found. This is illustrated in Figure 2. For more explanation on this part you can go to [16].The enumeration step is the power of this algorithm, which makes it different from other algorithms. The data structures used in the implementation of this step and the algorithmic idea makes it less time consuming. Also, having no limitation in the implementation of this step due to the size of motif is its advantage comparing to some algorithms like FANMOD or mfinder.

After discovering a sub-graph, involved as a match in the target network in order to be able to evaluate the size of each class according to the target network, Kavosh employs the Nauty algorithm [16] in the same way as the FANMOD does.

Generating random networks in CytoKavosh and Kavosh is similar to Milo's random model-switching operations. Generating random networks is an essential step in any motif discovery algorithm, as the concept of network motif is meaningful in comparing the frequencies of each sub-graph in the given network with which we except in random ones. By using switching operations, more restricted random networks are generated. The operation is applied on the edges of the input network repeatedly, until the network is well randomized. This switching operation is applied on the randomly chosen nodes of the network. By applying this switching operation repeatedly on the input network, an ensemble of random networks is generated.

CytoKavosh improves the CPU time and memory usage in comparison with other algorithms. It can also be employed for finding motifs of the sizes greater than eight, while most of the other algorithms have restriction on motifs with the size greater than eight. Besides, comparing with other algorithms, CytoKavosh has better performance for large networks.

The time complexity of the algorithm is *O*(*G*(*s*)×(*s* ^2^+*N*(*s*))), where *G*(*s*) is the number of sub-graphs of size *s* in the network, *N*(*s*) = *s*! is the complexity of Nauty in the worst case, and *s* ^2^is the time required in an embedded part of the classification (isomorphism testing) step of the algorithm (see ‘Results’ section). But the point is that the mentioned upper bound only occurs very seldom, if not in practice ‘Nauty’ will encounter the worst case only when the sub-graph is regular, which is a rare occasion in the biological networks with a scale-free nature. Thus, practically, the algorithm runs several orders of magnitude faster. The most important factor influencing and limiting the speed of CytoKavosh is the number of sub-graphs of size s of the network, *G*(*s*), which obviously might differ between two distinct networks of even the same size. So, the running time of the algorithm depends on the network for which the algorithm is being executed.

The upper bound of the memory required by CytoKavosh can be measured by formula *O*(2*^s^* ^∧2^+*s*×*MD* ^2^+*s* ^2^), where *MD* is the maximum degree of nodes in the given network. In practice, the size of the balanced binary tree used for isomorphism testing will never reach 2*^s^* ^∧2^ (the first term in the formula). Indeed, the size of this binary tree depends on *I*(*s*), the number of non-isomorphic classes of sub-graphs with size *s* found in the network, which indicates the number of leaves of the mentioned balanced binary tree. As our experiments indicate, *I*(*s*) is usually very less than its maximum possible amount, let's call it *I_m_*(*s*), which is yet very smaller than 2*^s^* ^∧2^. For example, when *s* = 4, then *I_m_*(*4*) = 199<<2^16^, but in our experiments the greatest *I*(4) observed in a network is 108. So the memory usage of CytoKavosh is mainly determined by *I*(*s*) in the given network.

With respect to above, as in other algorithms, it is the nature of a given network which determines the required processing and storage capacities for finding motifs of a specific size in that network. But what makes Kavosh different is the enumeration method it utilizes, which costs less when finding each sub-graph in the network. In other words, *G*(*s*) is multiplied by a smaller amount in Kavosh than other similar algorithms. Moreover, exploiting Nauty algorithm (as a fast algorithm for isomorphism testing) is another point of strength.

CytoKavosh uses Java for executing native Kavosh program, written in C++. CytoKavosh can also group the isomorphic sub-graphs by computing the canonical labeling. Native C++ code uses Nauty API for computing canonical labeling that makes the native code very fast. So, we decided to import these native C++ codes to make this plug-in as fast as the original program, written in C++. Table 1 demonstrates the running time of the execution of plug-in for some given sample networks. The transcriptional network of *E. coli* with 672 nodes and 1276 edges, a social network with 67 nodes and 182 edges, and an electronic network with 97 nodes and 189 edges are examined networks. As mentioned in the previous part, CytoKavosh can detect and compute motifs in any given networks with any given size; this characteristic makes this plug-in the best choice for finding motifs among other existing tools.

**Table 1:** Execution results of some sample networks, related to 4 to 7 motif sizes with 100 random network sizes.

Network	4-size motif	5-size motif	6-size motif	7-size motif
**E. coli**	2	16	149	1407
**Electronic**	0.45	2.37	11.8	73.11
**Social**	0.3	1.89	11.49	70

Rows indicate the running time (seconds) of the studied tool for each motif size.

**Figure 3:**
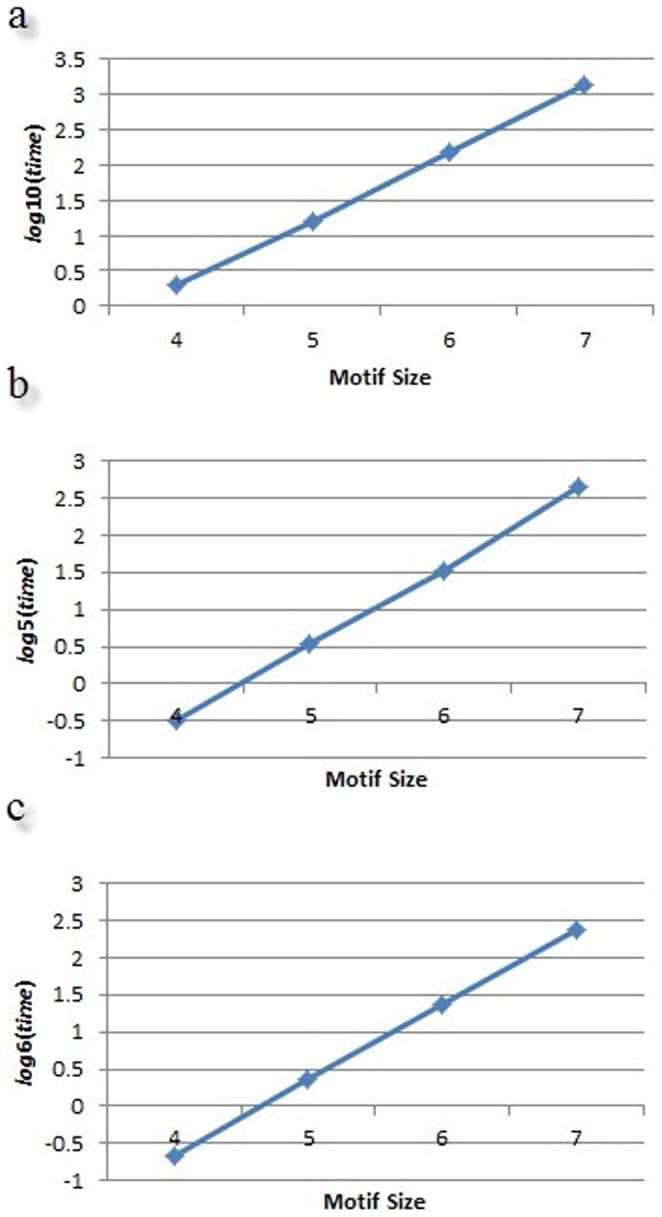
Exponential growth of processing time by motif size. Illustrated results are for (a) transcriptional network of *E. coli*, (b) Electronic network, and (c) Social network.

Furthermore, processing time is more important than memory in Kavosh and can limit the size of discoverable motifs. It is difficult to specify the exact relationship between the size of motif and the processing time, but roughly speaking based on our experiments, in Kavosh it is closer to an exponential relationship than other algorithms. This is illustrated in figure 3 for networks of table 1.

## Results

**Figure 4:**
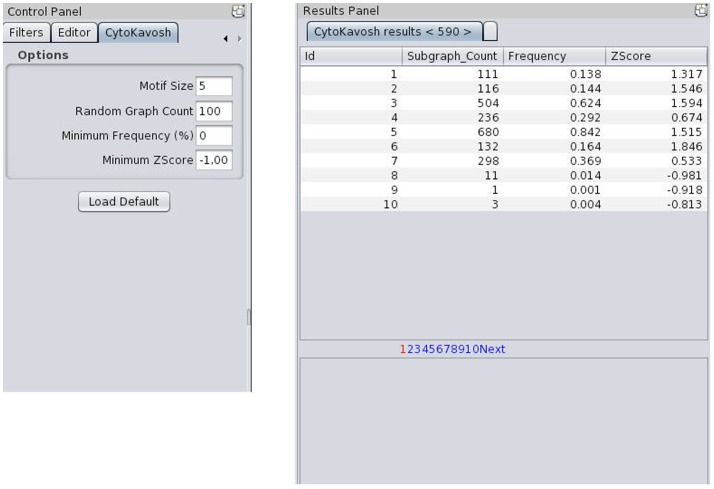
Control Panel of CytoKavosh, including the CytoKavosh control tab for getting input parameters. The right side of the figure shows the ‘results’ table panel after running the CytoKavosh for given input parameters. A table for each run of plug-in appears in the separate tab in ‘result’ panel. These tabs keep the results until finishing the plug-in. For larger sizes of the motifs, the number of detected motifs increases exponentially. So, the ‘results’ table can be explored page by page. The below panel shows the graphical representation of selected motif in the table.

The first step of running the plug-in is the loading of network into Cytoscape among loading dialogs. The input graph (directed or undirected) is build from loaded network. By choosing “CytoKavosh” sub menu from “Plug-ins” menu and by starting this plug-in, the program will start working. The next step, following loading of the network, is specifying the input parameters required by the program. These parameters are located in the separate control tab that is named as “CytoKavosh”. Figure 4shows the control panel including input parameters. Plugin can operate on any network format, supported by Cytoscape engine.

The size of motif to be discovered would be given by the user. This size for current version of plug-in can vary from 3 to 9 but the main algorithm supports any given number for motif size.

The significance of a sub-graph is evaluated by some measures such as its frequency, Z-Score and P-Value which are later described in details. Accuracy of these measures increases if the program generates more random networks. Results show that 100 random-networks is a good count to begin. When the size of motif increases, the total number of detected motifs increases exponentially. So, filtering the results on result attributes comes into necessary. For achieving this goal, there are two parameters for filtering results in the control tab.

There are two minimum thresholds for frequency and Z-Score, which can be given by the users for filtering results, according to their purpose. The calculated significance of each sub-graph in the network (like Z-score and P-value with respect to the generated random networks) can be calculated from frequency concept. CytoKavosh computes and analyses the network for finding motifs, in following steps:

### Sub-graph detection

CytoKavosh traverses the input network and finds all sub-graphs of a given size, which exist in the input network. Each sub-graph should be tested for its isomorphism class.

### Sub-graph isomorphism testing

During step 1, a binary tree is constructed. This tree holds non-isomorphic sub-graphs in different paths of the tree. Traversing this tree gives the adjacency matrix of each motif, and each path from root to a leave is related to one isomorphic class.

### Evaluation of a sub-graph

The number that is stored in the leaves of tree represents the number of matches of each sub-graph in the input network. As mentioned before there are three measures for evaluating an isomorphic class of sub-graphs. Each are described in details in the following.

Frequency for each sub-graph is calculated from the bellow-stated formula:
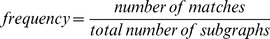



Statistical measures such as Z-Score and P-Value are very important factors for comparing sub-graphs. CytoKavosh gives Z-Score by generating random networks. One of the flexibility of this too is that the number of random networks is determined by user and it varies from 1 to 100. The Z-Score for a discovered sub-graph *G_P_* is calculated by the following formulas:
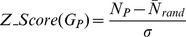



Where *N_P_* is the number which *G_P_* occurred in the input network, 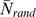 is the mean number which *G_P_* occurred in random networks and σ is standard deviation. The larger the Z-Score, the more the significance of the sub-graph.

The P-Value measure indicates the number of random networks in which a sub-graph *G_P_* occurred more often than in the input network, divided by the total number of random networks. The smaller P-Value, the more significant is the sub-graph.

Figure 4 shows the input parameters and output table for *E. coli* network.

For each execution of the program for given input parameters, there is a separate tab in the ‘Results’ panel, containing a table that sorts and classifies the detected motifs. Each row represents the attributes of a sub-graph (directed or undirected, depends on the input network) that is expressed as the motif. This sub-graph is a unique topological sub-graph in the whole given network.

**Figure 5:**
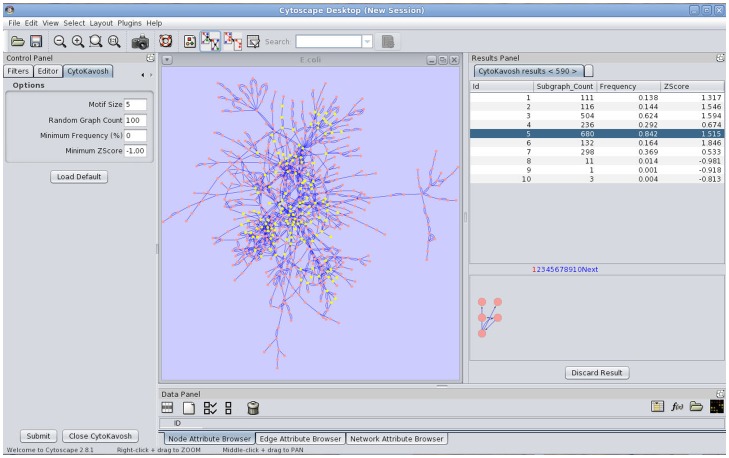
A sample of CytoKavosh ‘results’ table in which the motif size is 5 and the number of generated random networks is 100. All 590 motifs are listed in this picture in separate pages. Each page shows only 10 motifs. By clicking on each row (each motif), a graphical image of that motif appears in below panel. For exporting this view, we can right click on the row and export the motif in a separate network. For each running of CytoKavosh, there will open a new tab in ‘result’ panel, with the number shown in <> is the number of found motifs. In this sample, there are 590 motifs of size 5 for the transcriptional network of E. coli. Sub-graph count presents the number of sub-graphs of this motif type.

Attributes of a motif in the resulting table are frequency, Z-Score and the number of occurrences of that motif in the network. Sorting the attributes assists users to export knowledge from the results with graphical facilities deployed in the program. Showing the graphical representation of sub-graph, related to selected motif, is one of the graphical facilities. Figure 5 shows the graphical representation of selected motif in the bottom of Results' table.

User can select desired motif in the Results' table and view the occurrences of all of its sub-graphs in the main loaded network-graph. CytoKavosh highlights all sub-graphs of a selected motif in main graphical view of loaded network. Highlighting the sub-graphs in the main network helps the analysts to identify where each of these sub-graphs are occurred mostly in order to recognize the structural behavior of each part of the network. Figure 5 represents the ‘Results’ panel concluding from the running of CytoKavosh to find motifs with size 5 in the given network. All highlighted nodes are represented for selected motif in the ‘Results’ table and the sub-graph image of that motif are shown in the below-stated panel.

**Figure 6:**
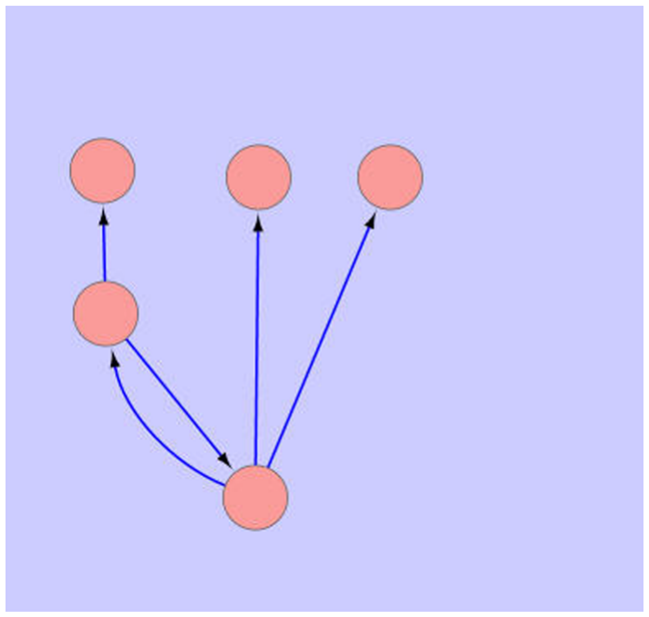
A sample of exported motif in a new network view.

With right clicking on a motif in the list, user can export the image of that motif in a separate network view in the Cytoscape. As the size of motif increases, the number of finding motifs, especially in large networks, increases too. Figure 6 represents the sub-graph of exported motif in a separate network view. User can save this network or use it in the Cytoscape like other networks.

## Experiments

For better understanding the advantages of CytoKavosh plug-in, we chose FANMOD software to compare and detect the motifs. Because the FANMOD is the most efficient tool according to time among other existing tools like MAVisto, Mfinder and MODA. So, the comparisons with other tools are just limited to FANMOD.

The main advantage of CytoKavosh plug-in over FANMOD is that CytoKavosh is based on Kavosh algorithm that operates over any given size of motif (more than 8) but FANMOD cannot detect up to 8 vertices of motifs. Table 2 shows the running time of two tools for *E. coli* network. It can be seen that the time usage of CytoKavosh is less than that in FANMOD in the equal size of motif. We limit the comparing part to one network, because there is well comparison in the main algorithm paper [16]. For more information about time and memory performance going to [16] is recommended.

**Table 2:** Comparison of the running times of two tools on the E. coli network.

	3	4	5	6	7	8	9
**CytoKavosh**	0.3	1.84	14.51	141.98	1374	13173	121110
**FANMOD**	0.81	2.53	15.71	132.24	1205.97	92566.1	-

Rows indicate the running time (seconds) of the studied tool for each motif size. 10 random networks are generated for this comparison.

Both tools can operate on directed and undirected graphs and both of them allow users to filter their results. But, another advantage of CytoKavosh is the graphical options to display the results. FANMOD can export the table of result for detected motifs in a dummy file, which does not allow us to carry out any modification or additional operation on it. On the other hand, CytoKavosh makes it possible for users to get an overview of occurrences of motifs within the whole network by highlighting them in the main network view.

There is not any other Cytoscape plug-in, specific for finding network motifs, based on the original term introduced by Milo [1]. CytoKavosh is the first plug-in, introduced by our research group, which can be further improved to cover other options to make it the best motif-analyzing tool.

## Conclusions

In Molecular Biology, there is a need to study interactions between biological elements like DNA, RNA, proteins or genes. To model these interactions, we adopt graph theory concepts: the biological elements that interact with others are represented by vertices and their interactions are represented by edges or arcs, depending on the type of interactions. We, hence, obtain a large graph called biological network. To extract pertinent knowledge from a biological network, we adopt several techniques, like network clustering [17], network modularity [17] and network motifs extraction [1]. Biological network motifs are of notable importance largely because they may reflect the properties relating to the biological functions. They play an important role in the analysis of biological networks since they are seen as the building blocks of these networks and shape their behaviors.

CytoKavosh is a flexible and extendable tool, used to analyze motifs in the biological networks. We believe that embedding the CytoKavosh in Cytoscape will further contribute to the establishment of Cytoscape as an integrated suite of tools for the analysis of biological networks. The high performance of CytoKavosh is achieved by dynamically linking the highly optimized C++ functions of the Kavosh program to the Cytoscape Java program, which makes CytoKavosh extremely suitable for the analysis of large biological networks. As Cytoscape continues to evolve, CytoKavosh evolves alongside it. The original algorithm is highly efficient and allows further extension. In particular, future versions can extend CytoKavosh towards the evaluation of motifs with larger sizes and also the parallelization of the algorithm for analyzing huge networks. Details of the main part of CytoKavosh algorithm are explained in Appendix 1.

## Availability

The tutorial, full package and associated examples are available at the following website: http://lbb.ut.ac.ir/Download/LBBsoft/CytoKavosh
